# Supplementary material for: Opportunities for decentralised solar power to improve reliability, reduce emissions and avoid stranded assets
Source: Nat Commun. 2025 Aug 28;16:8061. doi: 10.1038/s41467-025-62948-8 (PMC12394599; doi:10.1038/s41467-025-62948-8)
Supplement: Supplementary file 1 — Supplementary Information [file 41467_2025_62948_MOESM1_ESM.pdf]

Supplementary Information accompanying: Opportunities for decentralised solar power to improve reliability, reduce emissions and avoid stranded assets

Philip Sandwell[1,2,†], **Benedict Winchester**[1,2,3,†], **Shivika Mittal**[2,4], **Christos N. Markides**[3], **Hamish Beath**[5*], **and Jenny Nelson**[1,2**]

[1]Department of Physics, Imperial College London, London SW7 2AZ, UK

[2]Grantham Institute – Climate Change and the Environment, Imperial College London, London SW7 2AZ, UK

[3]Clean Energy Processes (CEP) Laboratory, Department of Chemical Engineering, Imperial College London, London SW7 2AZ, UK

[4]CICERO Center for International Climate Research, Oslo, Norway

[5]Centre for Environmental Policy, Imperial College London, London SW7 2AZ, UK

[*]hamish.beath16@imperial.ac.uk

[**]jenny.nelson@imperial.ac.uk

†These authors contributed equally to the work

Supplementary Note 1: Modelling electricity supply and demand in rural India

The community modelled aims to represent a typical rural community in Uttar Pradesh[1] and comprises 547 households and 66 enterprises. The average solar generation of 1 kW_p of installed PV capacity at the investigation location in Bahraich District, Uttar Pradesh (Supplementary Figure 1a), shows that solar power is typically available from 8:00–17:00 and there is comparatively lower generation, almost halving the hourly output, during the monsoon season between July and September. The relative availability of the grid is generated by modulating the recorded profile for Bahraich District to supply 13 hours per day (Supplementary Figure 1b) based on the average in Uttar Pradesh at the time of data collection being 12.5 hours per day[1]. The grid availability varies significantly both daily (with its lowest availability typically during the daytime) and seasonally (with its lowest availability between March and August).

Supplementary Figure 1c and d show the total load demanded by the community, composed of 547 households and 66 enterprises, and the percentage of the load from the enterprise activities respectively. These are derived from extensive household surveys described in Agrawal et al. (2019)[1] in which those authors describe a representative rural community in Uttar Pradesh and its energy demands. In this work we use these data (Supplementary Figure 1c and d, and Supplementary Table 1), to synthesise demand profiles reflective of daily and seasonal variations. The overall community demand (with an average of 761 kWh/day) is dominated by domestic energy uses (695 kWh/day), with a significant peak between 17:00–22:00 from demand for lighting, entertainment and fans. The commercial demand (66 kWh/day), including agro-processing machinery as well as lighting and appliances in shops, constitutes up to 25% of the total demand at any point in time and is concentrated in March, April, and October to December during the harvesting seasons for rice and wheat when the community milling machine is used the most[2]. The average electricity usage by households (38 kWh/month) and enterprises (30 kWh/month) found by Agrawal et al. (2019) and used here are very similar to those found in other studies[1,3] and correspond to most users having Tier 2 or 3 electricity access under the Multi-Tier Framework[4].

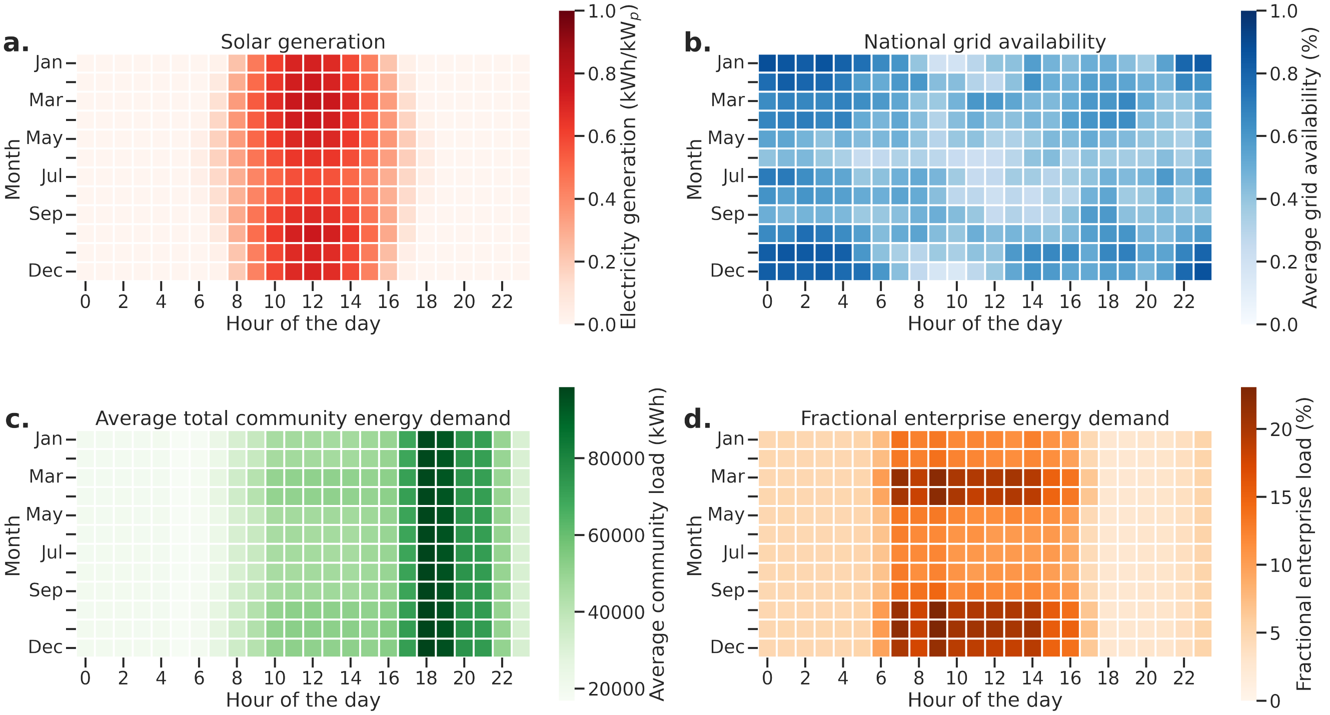

Supplementary Figure 1. Electricity supply and demand inputs to the modelling process: (a) solar generation (kW generation per kW_p), (b) probability of grid availability (shown for the average availability in Uttar Pradesh of 12 hours per day), (c) total community energy demand (761 kWh/day), and (d) proportion of energy demand from rural enterprises.

Supplementary Note 2: CLOVER model operation

Supplementary Figure 2 outlines the process of the model operation within CLOVER for the two scenarios: “grid prioritisation” and “local generation prioritisation.” Under the local-generation scenario, the load is considered, the available PV energy is subtracted followed by the available energy from the battery storage. If these are not sufficient, then energy is taken from the grid. Under the grid-prioritisation strategy, energy from the grid is used first to fully meet demand if available. If the grid is unavailable, energy from the PV system is used to meet demand, followed by power from the batteries.

Parameters which are included in the modelling framework when running simulations and optimisations of the energy system are given in Supplementary Tables 2 and 3 for the technical and costs-and-emissions inputs, respectively. These data were accurate at the time of writing, and references have been given where appropriate.

Supplementary Note 3: Grid subsidy schemes

Supplementary Figure 3 shows the percentage difference in the LCUE of systems optimised under an unsubsidised grid tariff of 0.10 \$/kWh compared to those optimised under a subsidised tariff of 0.06 \$/kWh. The colour indicates the fraction of the electricity for the optimised system that was sourced from the grid. The results show an overall increase in the LCUE of the systems, with those systems that rely more heavily on the grid experiencing a greater increase in their LCUE, up to a 4 cents difference (25%) for systems that rely entirely on the grid as a source of power.

Supplementary Table 1. Ownership, usage and power ratings of a representative rural community in Uttar Pradesh derived from the findings of Agrawal et al. (2019)[1]. The community is composed of 547 households, 66 enterprises and one milling machine for processing crops, the use of which varies seasonally. The power demand and usage of the same appliance type in domestic and commercial settings can vary and is based on the findings of that empirical study.

Demand type	Appliance	Number	Ownership (%)	Usage (hrs/day)	Power (W)
Households (547)	LED bulb	1363	249	4	8
	Incandescent bulb	456	83	3	97
	Phone charger	432	79	3	5
	Table fan	153	28	8	62
	Ceiling fan	291	53	13	72
	Television	190	35	5	36
	Refrigerator	38	7	11	41
	Dessert cooler	31	6	9	224
	Iron	30	6	1	858
	Home water pump	22	4	1	1265
Enterprises (66)	LED bulb	46	69	4	8
	Incandescent bulb	6	1	3	103
	Table fan	18	28	7	72
	Ceiling fan	26	40	8	70
	Refrigerator	9	13	9	41
	Laptop	5	7	6	50
	Printer	3	5	5	350
	Milling machine	1	–	2–7	7460

Supplementary Table 2. Model inputs used for technical specification of mini-grid systems. For the year of values given, see reference year.

Item	Value	Unit	Reference	Notes
Battery depth of Discharge	70	%	[5]	LFP battery chemistry
Battery leakage	0.004	% per hour	[5]	LFP battery chemistry
Battery cycle lifetime	2500	Cycles	[5]	LFP battery chemistry
Battery roundtrip efficiency	86	%	[5]	
Battery lifetime loss	80	%	[6]	
Battery c-rate in	0.2		[5]	
Battery c-rate out	0.2		[5]	
Transmission efficiency AC	95	%		
DC to AC conversion	95	%		
DC to DC conversion	95	%	[7]	
Inverter lifetime	10	Years	[8]	
Solar PV lifetime	20	Years		
Solar PV azimuth	180	degrees		
Solar PV tilt	29	degrees		

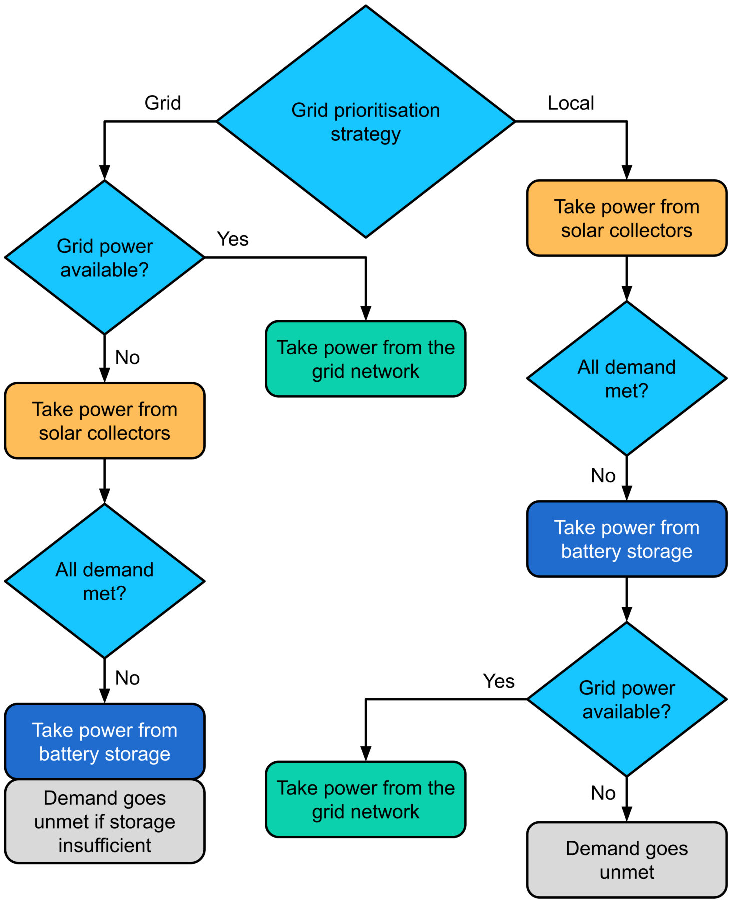

Supplementary Figure 2. Flowchart showing the operation of the energy-balance model under both "grid-prioritisation" and "local-generation" scenarios. If power from the national-grid network is prioritised (left branch), provided that grid power is available, all of the demand is taken from the national-grid network to meet the local loads. If the national-grid network isn't available, then power is taken from any solar collectors; namely, photovoltaic (PV) panels. If the installed PV panels were unable to meet demand (*e.g.*, at night), then batteries are discharged to meet any additional demand. Any demand that remains at the end of this process remains unmet. If local power is prioritised, then power is taken from the national-grid network as a last resort: power is taken first from PV panels, then from battery storage, with the national-grid network, if available, providing any demand that remains unmet. Prioritising local power reduces a mini-grid system's reliance on the national-grid network, generally resulting in a lower carbon footprint per unit electricity consumed (its emissions intensity) as national-grid networks have higher quantities of embedded carbon and use carbon-intensive fossil fuel–based technologies as part of their energy mix. However, prioritising locally-generated electricity first and national grid–sourced power second can result in a lower overall reliability: if power is drawn down from battery storage, or is consumed directly from a solar PV array, when the national-grid network is available, then, at a later time, when the national-grid network is unable to provide electricity to the system, demand may go unmet. If grid-sourced power were prioritised, then locally-generated electricity could be stored during hours when grid-sourced power was available for use at later times. With an unreliable national-grid network, a grid-prioritisation strategy often results, therefore, in higher carbon emissions and higher levels of reliability than systems that prioritise locally-generated power.

Supplementary Table 3. Model inputs used for the economic and GHG assessment of mini-grid systems. Cost values are given in 2022 dollars and for the year 2022; the year of other values varies, depending on data availability, see year of reference.

Item	Cost/Value	Unit	Ref	GHGs	Unit	Ref
Solar PV Panels	320	$/kWp	[9]	790*	kgCO2/kWp	[10]
Solar PV O&M	1	% of CAPEX p.a	[9]	—	—	—
PV Inverter	163	$/kW	[11]	124*	kgCO2/kWp	[14]
Battery Storage	250	$/kWh	[9]	110	kgCO2/kWh	[15]
Battery O&M	2	% of CAPEX p.a	[9]	—	—	—
Balance of System	100	$/kWh	[11]	134	kgCO2/kWp	[14]
Discount Rate	8	% per annum	[13]	—	—	—
Meter, Connection & Distribution Cost	225	$ per household	[5,9]	—	—	—
General O&M	2400	$ p.a.	[11]	—	—	—
Grid Tariff	0.06	$ /kWh	[12]	—	—	—
Power equipment & integration	256	$ /kW	[9]	—	—	—

*Based on manufacture in China.

Supplementary Note 4: Asset stranding

Supplementary Figure 4 shows the total and additional service hours provided for systems that undergo "asset stranding," whereby the reliability of the grid changes and systems are now either undersized to meet demand (in the event of a reduction in grid service hours) or are stranded and under-utilised (in the event of an increase in grid service hours). Supplementary Figure 4a shows the total number of these service hours as a heatmap, with systems in the centre column (13 hours) operating in the regime of grid electricity that they were optimised under, and those to the left and right operating under a reduced or increased grid service respectively. Visible to the left is a reduction in the total number of service hours provided by these assets as they are no longer correctly sized to meet the electricity requirements of the system. Systems sized for a lower number of service hours are most affected by this, with systems sized for 14 hours of service supplying as little as 0.79, 0.82 and 0.88 hours of service beyond the grid when the grid availability is reduced to 0, 1 and 2 hours of availability. This is due to this system having only a small number of installed assets as only 1 additional hour of service was originally required.

To the right of Supplementary Figure 4a., increases in the grid service hours result in a greater number of hours of service being supplied overall, with assets still able to supply hours beyond those provided by the grid. In Supplementary Figure 4b., the change in the service hours provided is visible. It is clear from the figure that systems sized to meet the largest demand are least affected by the reduction in grid service hours. Less clear, but visible in the figure, is that those systems sized to meet an intermediate number of hours (17–20 hours of service) provide the greatest number of additional hours beyond those provided by the grid, with over 3 hours of additional service provided in the most extreme case. These systems hence provide the greatest resilience against asset stranding as they provide the greatest number of service hours beyond those provided by the national grid which can be utilised and sold to the community as a more reliable service to generate revenue.

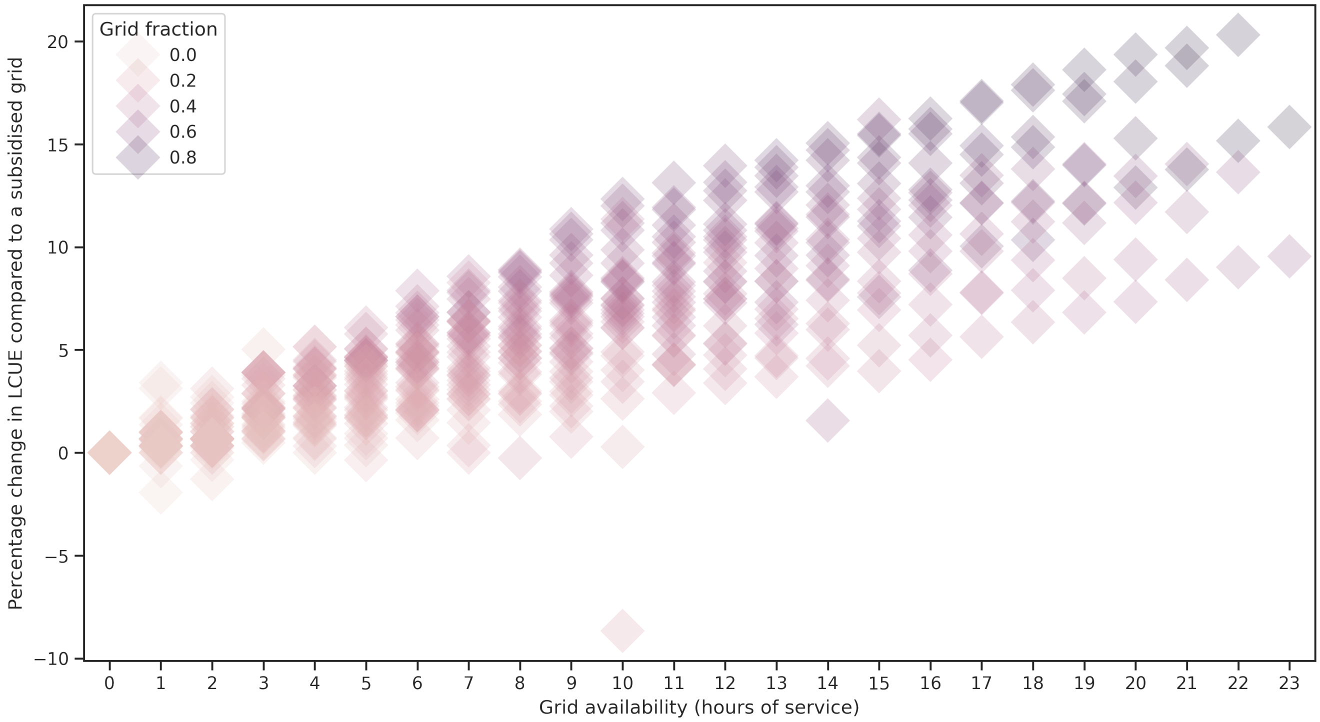

Supplementary Figure 3. The electricity in Uttar Pradesh is currently subsidised for consumers at a rate of 0.06 $/kWh. The effect of changing the rate for grid-sourced electricity to the unsubsidised value of 0.10 $/kWh is shown, with the percentage change in the overall LCUE of the system when removing the subsidy plotted against the average availability of the grid. The colour represents the fraction of electricity that was sourced from the grid for a given system.

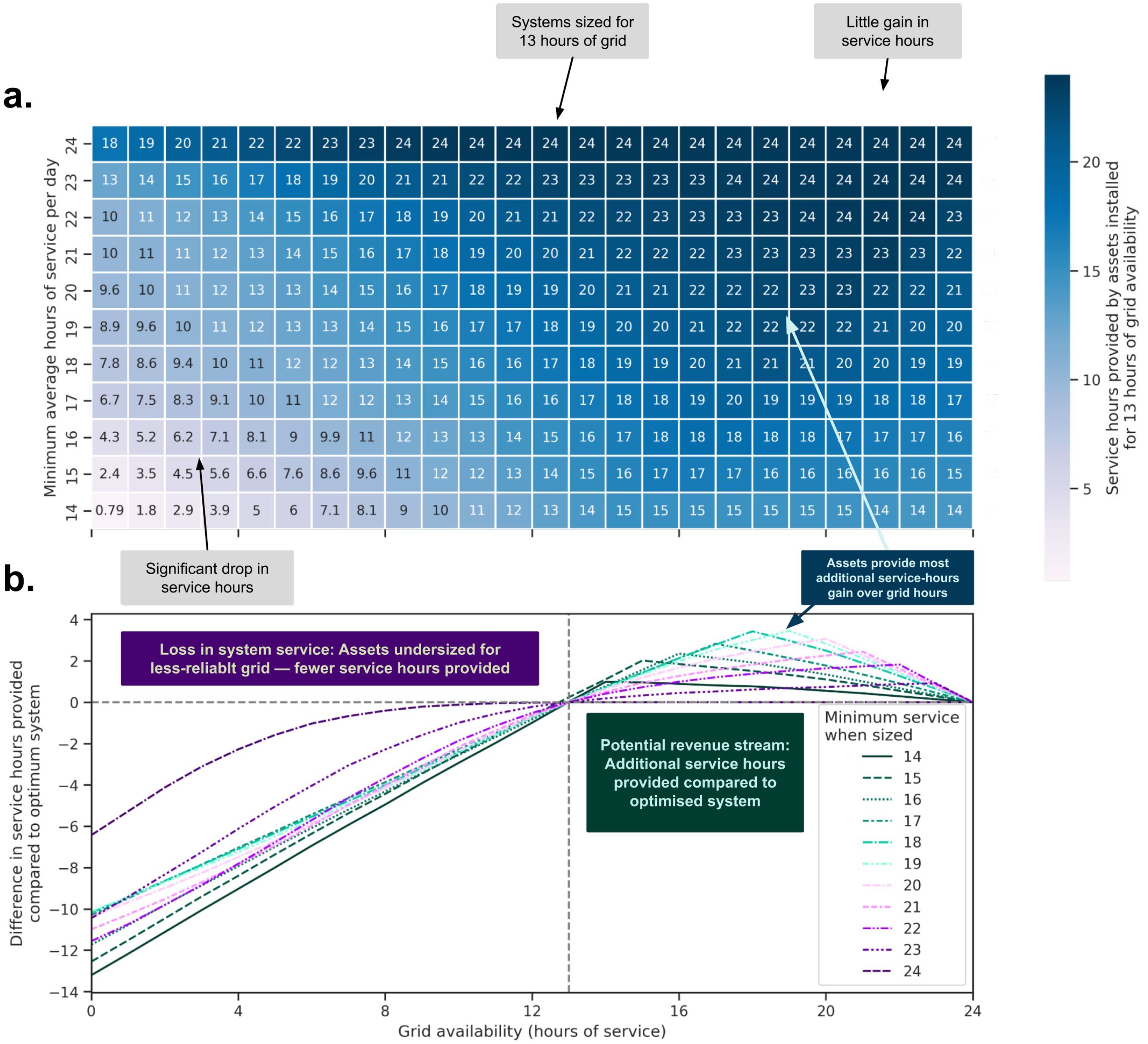

Supplementary Figure 4. Service hours provided for systems initially sized to meet a given number of service hours (shown on the y-axis) for 13 hours of grid-electricity availability when the reliability of the grid changes, with increasing reliability shown to the right and decreasing to the left: (a) heatmap, showing the total number of hours for which service was provided, and (b) plot of the difference between the service hours provided by the system sized with 13 hours of grid electricity available and the optimised system as the grid reliability changes. Each line represents a system sized to meet a different number of service hours with 13 hours of grid electricity available.

Supplementary Note 5: Asset-stranding scenarios

Presented in the main manuscript (in Figure **??**) is the impact of a changing user base (demand profile) coupled with improvements in the reliability of the national-grid network. The scenarios presented in the main manuscript are representative of this uncertain space: whilst increases in the national-grid availability are likely to reduce demand as customers switch away to an equally-reliable and more-affordable service, the exact relationship between these two factors is difficult to predict. As such, the scenarios presented in the main manuscript (detailed in Table **??**) are representative of potential combinations of these factors.

Figures 5 and 6 show the impact of the grid reliability and changing user-base on the utilisation rate of the local renewable assets, respectively, with the two effects here decoupled. The data show the general trend that a reduction in demand, or an increase in reliability of the grid, reduces the asset utilisation—these assets are effectively stranded as they are under-utilised (un-utilised in the most extreme cases of 100% demand drop and 24 hours of grid availability, respectively) and contribute unnecessary costs to the system.

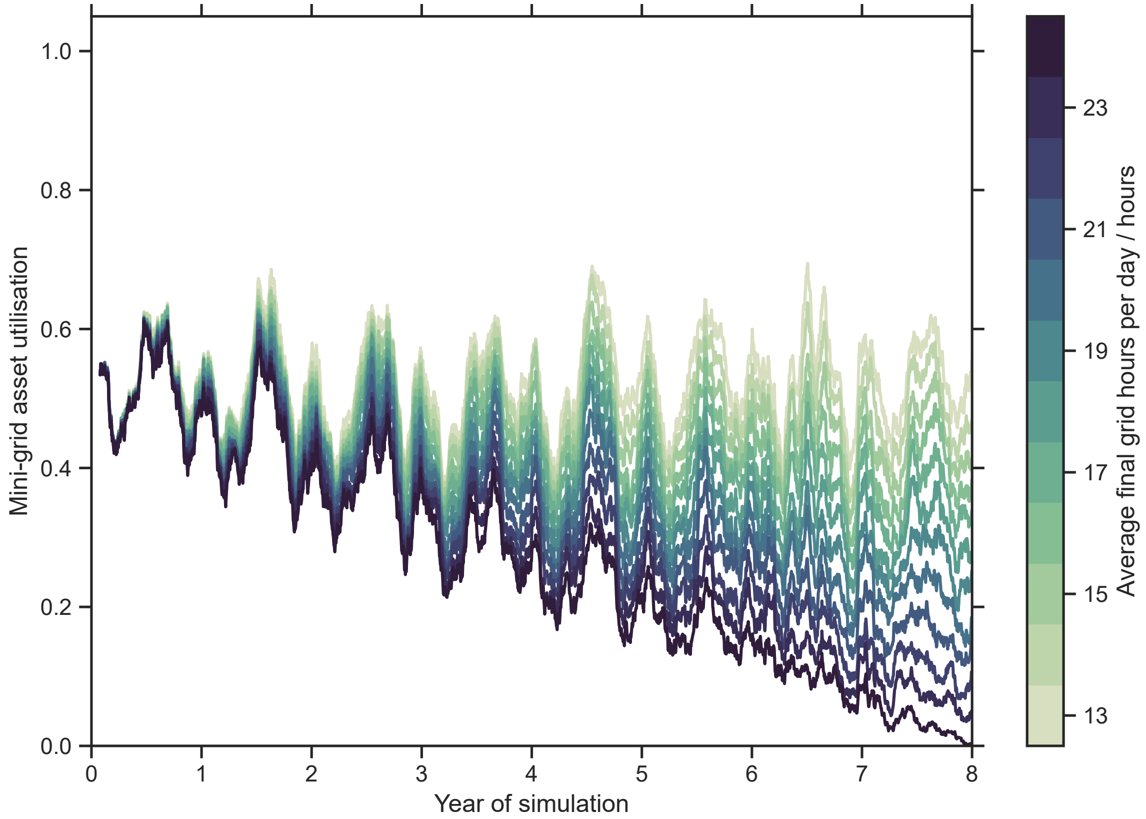

Supplementary Figure 5. The utilisation of local renewable assets subject to asset stranding as the reliability of the grid improves over the modelling period. The data show the utilisation rate (taken as a 30 day average) for scenarios ranging from no improvement in the reliability (a final reliability of 13 hours of service per day) to a fully-reliable grid (24 hours of service per day). Assets were initially installed to meet 16 hours of demand (*i.e.*, three additional hours of service beyond those that the national grid was able to provide).

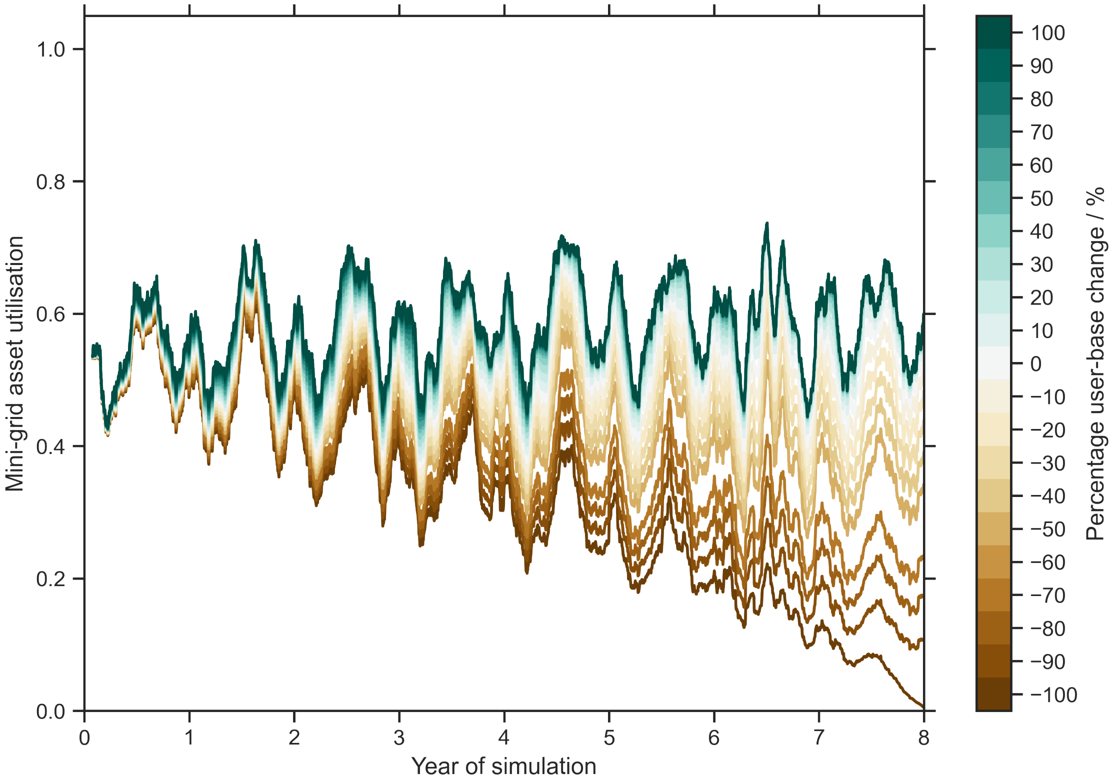

Supplementary Figure 6. The utilisation of local renewable assets subject to asset stranding, or improved utilisation, as the size of the demand changes over the modelling period. Increases in demand are shown with blue-green hues whilst reductions in demand are shown in orange-brown hues, with these resulting in increases and decreases in the utilisation of the local assets, respectively.

Where the demand increases, slight increases in utilisation are visible, but the limited storage capacity installed means that the local renewable assets are unable to provide much more power during night-time hours. Further, the national grid's output capacity (when available) is not capped, and so, when operating under a grid-prioritisation strategy (as shown) the grid is able to meet increases in demand when available.

Supplementary Note 6: Imposition of a carbon tax

Presented in the main manuscript are results which investigate the likely costs and emissions of installed mini-grid systems aiming to provide certain levels of service beyond that provided by the national-grid network. The optimisations undertaken aimed to produce system configurations which minimised the LCUE (see Equation **??**) subject to a minimum service requirement beyond the electricity provided by the national-grid network. Results surrounding the emissions intensity of the systems (Figure **??**d) hence show the emissions intensity of electricity generated by systems which aimed to minimise the LCUE but which may not have the lowest carbon intensity of those installed.

A common means of combining these metrics is through the imposition of a carbon tax or carbon price, thereby placing an economic cost on the carbon emissions produced. Given that investment will be needed for India to decarbonise its national-grid network, there will be a cost associated with the decrease in carbon intensity which we have assumed in our modelling. Figure 7 shows the impact of passing on this investment cost on the fraction of electricity sourced from the national-grid network, where the cost is incorporated as an increase in the cost of grid-sourced electricity and the effective carbon price used is 24.77 \$/tonne-CO_2eq taken as the average cost in the AR6 decarbonisation pathways[16].

The data show that systems which were sized to provide a relatively large additional service beyond the national-grid network experienced only a moderate shift away from grid-sourced electricity under the imposition of a carbon tax, up to around 1% for systems requiring 20 or more additional hours of provision, whilst those which provided limited service improvements showed the greatest shift away from the grid (shifts of 15–17% for systems sized to provide up to four additional hours of service beyond the grid). The data also show that the shift away from the national-grid network was most pronounced when the national-grid was least available, with shifts of up 10–16.5% (and 1.8–6.5% on average) for grid availabilities of up to 10 hours of service per day compared to shifts of less than 2% for grid availabilities beyond this.

These data make sense insofar as the imposition of a carbon tax on electricity sourced from the national-grid network makes using grid-sourced electricity more expensive, in a similar way to removing the grid subsidy (Supplementary Note). When the grid is relied upon heavily (such that local renewable assets provide little service), then this increase in cost has the greatest impact, shown by the lighter green hues having the greatest fractional decrease on the left of the figure for low hours of grid service.

When the grid is available for the majority of the hours of the day, then increasing the cost of the grid has limited impact on the system as installing local renewable assets is not as cost effective due to the prevalence of grid-sourced electricity and so, even with an increase in the cost of grid-sourced electricity, systems still rely on the grid (shown in the linearly-increasing trend across the figure). Where significant assets have already been installed (darker hues), the imposition of a carbon tax has little

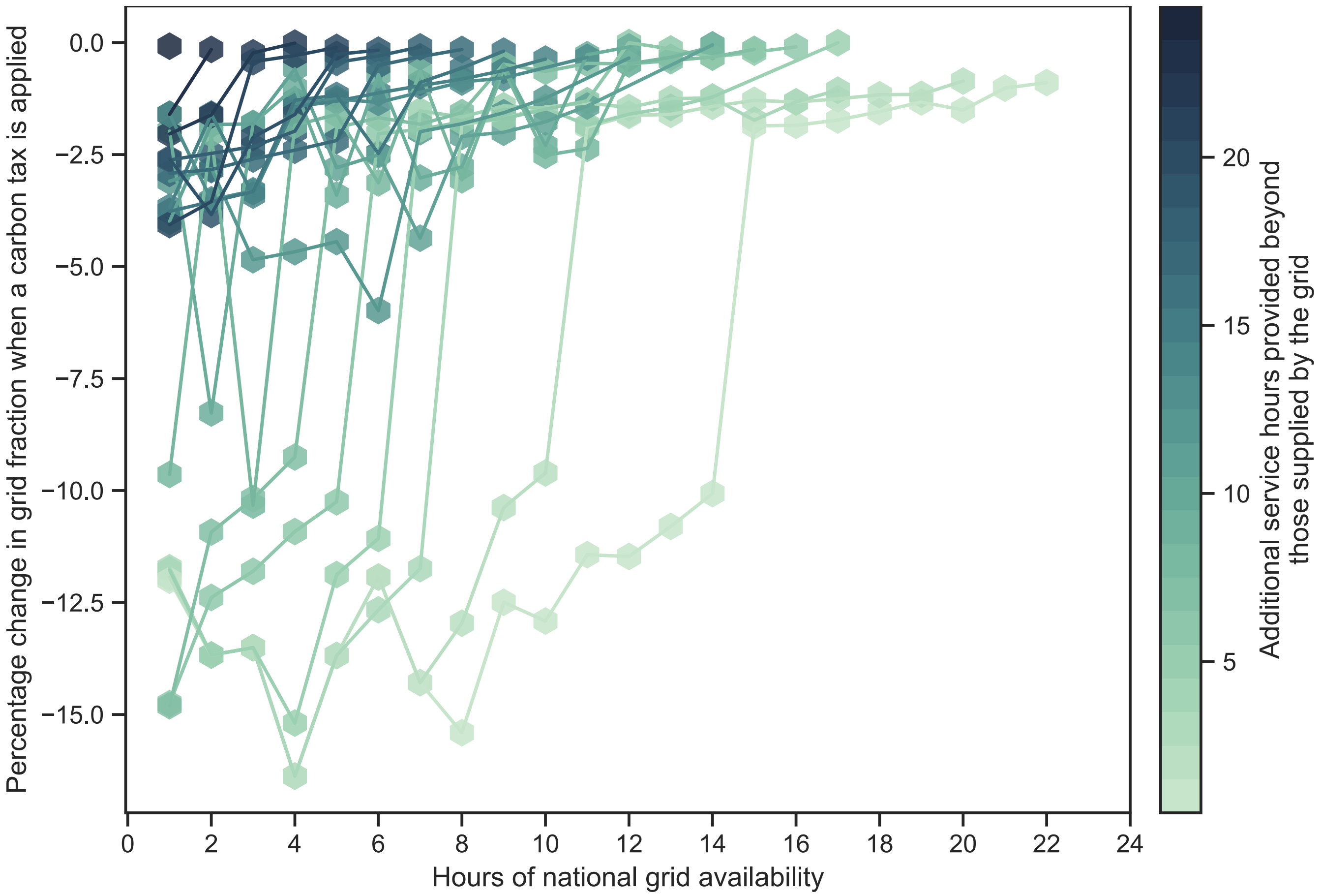

Supplementary Figure 7. The percentage change in the amount of electricity sourced from the national-grid network for systems prioritising locally-generated electricity under the imposition of a carbon tax. Systems which are sized to provide more additional service beyond the national-grid network are shown with darker hues whilst those which are sized to provide fewer additional service hours are shown in lighter hues.

impact on the system because the installed local assets already supply a significant fraction of the electricity consumed within the community making them resilient to increases in the grid cost.

Taken together, the data in Supplementary Figure 7 show that the imposition of a realistic carbon tax has the most effect for systems which are small in capacity (providing limited hours of service beyond the national grid) when the national grid is available for only a few hours of the day, where installing a small number of additional local renewable assets can reduce the reliance on the grid and provide cheaper electricity. It should be noted that no carbon tax was applied in this analysis for the emissions embedded in the locally installed renewable assets.

Any metric which aims to link costs and emissions is limited as, ultimately, they are separate measures of the impact of a system. A carbon tax based on the AR6 scenarios aims to capture the likely costs of decarbonising India's national-grid network. However, it is unlikely that all of these costs will be passed onto end consumers, meaning that the carbon tax results presented in Figure 7 represent an upper-bound estimate of the shift which could occur should these costs be passed on in-full to end consumers.

Supplementary References

1. Agrawal, S., Bali, N. & Urpelainen, J. Rural electrification in India: Customer behaviour and demand. Smart Power India & Initiative for Sustainable Energy Policy. Available at: https://www.rockefellerfoundation.org/report/rural-electrification-india-customer-behaviour-demand/ (2019).
2. Ministry of Agriculture and Farmers Welfare, Government of India. Crop calendar. Available at: https://nfsm.gov.in/nfmis/rpt/calenderreport.aspx (accessed 18 July 2019).
3. Chindarkar, N. & Goyal, N. One price doesn't fit all: An examination of heterogeneity in price elasticity of residential electricity in India. *Energy Econ.* **81**, 765–778 (2019), DOI: https://doi.org/10.1016/j.eneco.2019.05.021.
4. Bhatia, M. & Angelou, N. Beyond connections: Energy access redefined. ESMAP, World Bank. Available at: https://openknowledge.worldbank.org/handle/10986/24368 (2015).
5. Beath, H. *et al.* The cost and emissions advantages of incorporating anchor loads into solar mini-grids in India. *Renew. Sustain. Energy Transit.* **1**, 100003 (2021), DOI: https://10.1016/J.RSET.2021.100003.
6. Han, X. *et al.* A review on the key issues of the lithium ion battery degradation among the whole life cycle. *eTransp.* **1**, 100005 (2019), DOI: https://10.1016/J.ETRAN.2019.100005.
7. Pang, H., Lo, E. & Pong, B. DC electrical distribution systems in buildings. In *Proc. 2nd Int. Conf. Power Electron. Syst. Appl.* 115–119 (IEEE, 2006), DOI: https://doi.org/10.1109/PESA.2006.343082.
8. Sangwongwanich, A., Yang, Y., Sera, D. & Blaabjerg, F. Lifetime evaluation of grid-connected PV inverters considering panel degradation rates and installation sites. *IEEE Trans. Power Electron.* **33**, 1125–1236 (2018), DOI: https://10.1109/TPEL.2017.2678169.
9. Baldi, D., Moner-Girona, M., Fumagalli, E. & Fahl, F. Planning sustainable electricity solutions for refugee settlements in sub-Saharan Africa. *Nat. Energy* **7**, 369–379 (2022), DOI: https://10.1038/s41560-022-01006-9.
10. Elementa & Willmott Dixon. Whole life carbon of photovoltaic installations. Technical Report - February 2022. https://www.willmottdixon.co.uk/asset/17094 (2022).
11. Beath, H. *et al.* Maximising the benefits of renewable energy infrastructure in displacement settings: Optimising the operation of a solar-hybrid mini-grid for institutional and business users in Mahama Refugee Camp, Rwanda. *Renew. Sustain. Energy Rev.* **187**, 113058 (2023), DOI: https://doi.org/10.1016/j.rser.2022.113142.
12. Uttar Pradesh Electricity Regulatory Commission. 1 unit electricity cost in UP in 2024. https://housing.com/news/what-is-the-1-unit-electricity-cost-in-up/ (2023).

13. Tongia, R. Microgrids in India: Myths, misunderstandings, and the need for proper accounting. https://www.brookings.edu/research/microgrids-in-india-myths-misunderstandings-and-the-need-for-proper (2018).

14. de Wild-Scholten, M. J. Energy payback time and carbon footprint of commercial photovoltaic systems. *Sol. Energy Mater. Sol. Cells* **119**, 296–305 (2013), DOI: https://doi.org/10.1016/J.SOLMAT.2013.08.037.

15. Peters, J. F. *et al.* The environmental impact of Li-Ion batteries and the role of key parameters – A review. *Renew. Sustain. Energy Rev.* **67**, 491–506 (2017), DOI: https://doi.org/10.1016/j.rser.2016.08.039.

16. Byers, E. *et al.* AR6 Scenarios Database. https://doi.org/10.5281/zenodo.5886912 (2022).
